# Selective inhibition of TLR2 stimulates the maturation of oligodendroglial progenitor cells to oligodendrocytes

**DOI:** 10.1515/nipt-2026-0021

**Published:** 2026-09-02

**Authors:** Sudipta Chakrabarti, Malabendu Jana, Sukhamoy Gorai, Kalipada Pahan

**Affiliations:** Division of Research and Development, Jesse Brown Veterans Affairs Medical Center, Chicago, IL, USA; and Department of Neurological Sciences, Rush University Medical Center, Chicago, IL, USA; Division of Research and Development, Jesse Brown Veterans Affairs Medical Center, Chicago, IL, USA; and Department of Neurological Sciences, Rush University Medical Center, Chicago, IL, USA; Division of Research and Development, Jesse Brown Veterans Affairs Medical Center, Chicago, IL, USA; and Department of Neurological Sciences, Rush University Medical Center, Chicago, IL, USA; Division of Research and Development, Jesse Brown Veterans Affairs Medical Center, Chicago, IL, 60612, USA

**Keywords:** remyelination, oligodendroglial progenitor cells, oligodendrocytes, TLR2, multiple sclerosis

## Abstract

It is important to describe new technologies for overcoming the barrier and promoting remyelination because many axons remain demyelinated in the CNS of multiple sclerosis (MS) patients. Recent studies indicate that LR2 is a negative regulator of maturation of oligodendroglial progenitor cells (OPCs) to oligodendrocytes (OLs) and that TLR2 is present in human MS chronic lesions. However, until recently, there was no specific inhibitor of TLR2. Therefore, we have engineered a peptide corresponding to the TLR2-interacting domain of MyD88 (TIDM) that specifically inhibits TLR2, but not other TLRs. This study underscores the importance of wild type TIDM (wtTIDM) peptide in promoting the maturation of OPCs to oligodendrocytes. The expression of different maturation markers including PLP, MBP and MOG increased in cultured OPCs in response to wtTIDM, but not mTIDM. While investigating mechanisms, we found that wtTIDM peptide increased the maturation of OPCs isolated from TLR4^−/−^ mice, but not TLR2^−/−^ mice, indicating that wtTIDM requires the association with TLR2, but not TLR4, to exhibit this new function. Finally, as evident from double label immunohisto-chemistry and Western blotting, intranasal administration of wtTIDM, but not mTIDM, was capable of increasing OPC maturation and promoting remyelination in the corpus callosum of cuprizone-intoxicated mouse model of demyelination. These results indicate that wtTIDM stimulates the maturation of OPCs via TLR2 and that intranasal wtTIDM may be further evaluated preclinically for stimulating remyelination.

## Introduction

Multiple sclerosis (MS) is the most common demyelinating disease in humans and until now, no approved therapy reliably restores remyelination in MS [1–4]. In the injured CNS, the remyelination is initiated by a special subset of endogenous stem cells called oligodendroglial progenitor cells (OPCs), which arise from the ventricular zone during early development to proliferate and migrate into the different developing areas of the brain, where they differentiate into myelin-forming oligodendrocytes (OLs) [5, 6]. Despite having this endogenous machinery, many axons remain demyelinated in the CNS of MS patients. Therefore, delineation of new drugs/targets for overpowering the blockade and endorsing remyelination is an important area of research.

Toll-like receptors (TLRs) assist as vital links between innate and adaptive immune responses mostly by responding to bacteria, bacterial products, viruses, viral products, and flagellin [7–9]. Although in the beginning, TLRs were supposed to be present in cells of only myeloid origin, recent studies indicate the presence of TLRs in non-myeloid cells as well [10, 11]. For example, OPCs are rich in TLR2 [11, 12]. It has been demonstrated that TLR2 is present in human MS chronic lesions and that TLR2 is a negative regulator of OPC maturation [12]. According to Sloane et al.[12], genetic knockdown of either TLR2 or its downstream partner MyD88 enhances remyelination in an animal model of demyelination. However, gene knockdown of either TLR2 or Myd88 is not a therapeutic option. Since there was no specific inhibitor of TLR2, to target induced TLR2 from therapeutic angle, we have engineered a peptide corresponding to the TLR2-interacting domain of MyD88 (TIDM) that binds to the BB loop of only TLR2, but not other TLRs [9]. Accordingly, the wild type (wt) TIDM peptide selectively dissociates the interaction between TLR2 and MyD88 to inhibit the activation of only TLR2, but not other TLRs [8, 9].

Here, we establish that wtTIDM peptide stimulates the maturation of OPCs to OLs and that intranasal administration of wtTIDM peptide promotes remyelination in the corpus callosum of cuprizone-intoxicated mouse model of MS. Furthermore, by using OPCs isolated from TLR2^−/−^ and TLR4^−/−^ mice, we also demonstrate that wtTIDM peptide requires the interaction with TLR2, but not TLR4, for increasing the maturation of OPCs. Together, this study highlights wtTIDM as a prospective intranasal agent to promote remyelination in MS and other demyelinating disorders.

## Materials and methods

### Animals

Animal maintaining and experiments were in accordance with National Institute of Health guidelines and were approved (protocol ID: 22–19) by the Institutional Animal Care and Use committee of the Jesse Brown VA Medical Center, Chicago, IL. Mice were housed in ventilated micro-isolator cages in an environmentally controlled vivarium (7:00 A.M./7:00P.M. light cycle; temperature maintained at 21–23 ° C; humidity 35–55 %). Animals were provided with standard mouse chow and water *ad libitum* and closely monitored for health and overall well-being daily by veterinary staff and the investigator. This study was not preregistered. *C57BL/6J* (RRID: IMSR_JAX:000664), *TLR2*^−/−^ (RRID: IMSR_JAX:004650) and *TLR4*^−/−^ (RRID: IMSR_JAX:029015) mice were obtained from Jackson Laboratory, Bar Harbor, ME, USA.

### Reagents

Molecular Biology grade agarose (RRID: 1613102) was purchased from Bio-Rad (Hercules, CA). DMEM/F-12 (RRID: 10–092-CV), Hanks’ balanced salt solution (RRID: 21–022-CM), l-glutamine (RRID: 25–005-CI), 0.05 % trypsin (RRID: 25–051-CI), and antibiotic/antimycotic (RRID: 30–004-CI) were obtained from ThermoFisher (Waltham, MA). Fetal bovine serum or FBS (RRID: EF-0500-A) was obtained from Atlas Biologicals (Fort Collins, CO). Alexa-fluor 488 donkey anti-goat (RRID: 705-546-147), Alexa-fluor 647 donkey anti-rabbit (RRID: 711-605-152), and Alexa-fluor 488 donkey anti-mouse (RRID: 715-545-150) secondary antibodies used in immunostaining were obtained from Jackson ImmunoResearch (West Grove, PA). DAPI dihydrochloride (DAPI) was purchased from Millipore-Sigma (Burlington, MA).

### TLR2-interacting domain of MyD88 (TIDM) peptides

TIDM peptides (>99 % pure) were synthesized in the custom peptide synthesis facility of Genscript (Piscataway, NJ). TIDM peptides contain the Antennapedia homeodomain (lower case) and MyD88 (upper case) segments [8, 9, 13]. Positions of A→W and K→D mutations are underlined for TIDM peptides:

Wild type (wt) TIDM: drqikiwfqnrrmkwkkPGAHQK. Mutated (m) TIDM: drqikiwfqnrrmkwkkPGWHQD.

### Cuprizone feeding

Cuprizone-containing chow (0.2 % w/w) diet (RRID: TD.140803) was purchased from Envigo (Indianapolis, IN, USA). Eight-weeks-old *C57BL/6J* (RRID: IMSR_JAX:000664) male mice were provided cuprizone chow for 5 weeks.

### Intranasal treatment of *cuprizone-intoxicated mice* with TIDM peptides

Wild type (wt) TIDM and mutated (m) TIDM peptides were solubilized in normal saline in such a way that each mouse receives 0.1 mg/kg of body weight TIDM peptide in 2.5 μL of saline. After 5 weeks of cuprizone feeding, mice were given regular chow and treated with TIDM peptides intranasally (2.5 μL in one nostril) every day for a total of 21 days. While the right nostril was used for treatment one day, the left one was used the next day. Since TIDM peptides were solubilized in saline, one group of cuprizone-insulted mice also received 2.5 μL of saline as vehicle control. Mice were hold in supine condition while administering TIDM solutions [8, 9, 13]. Treatment started daily at 10 AM and continued for 21 days. During treatment, no mouse either reached the moribund stage or died. After 21 d of treatment, mice were anesthetized with ketamine/xylazine injectables followed by perfusion with PBS and paraformaldehyde. Animals were deeply anesthetized and transcardially perfused.

### Isolation of mouse OPCs

OPCs were isolated from P1-P2 neonatal pups as described before [14–16] with modifications. Briefly, the brain tissues from the pups were pooled together and placed together in the DMEM/F-12 media supplemented with 10 % heat-inactivated fetal bovine serum. Cells were dissociated by trituration, and single cell suspension was plated in poly-d-lysine (RRID: P0899; Sigma) pre-coated flasks containing complete DMEM/F12 media. On the 9th day, flasks were shaken for 2 h at 240 rpm to remove microglia from the rest of the cells. Then, on the 11th day, flasks were shaken overnight at 180 rpm, followed by 2 h shaking at 240 rpm to isolate OPCs. In order to remove microglia and astrocytes from OPCs, suspended cells were allowed to adhere on uncoated flasks for 1 h. Non-adherent cells were centrifuged at 1,000 rpm for 10 min and plated on experimental wells, plates or coverslips in the presence of OPC medium [DMEM containing 4 mM l-glutamine (RRID: 25–005-CI), 1 mM sodium pyruvate (RRID: 11360070L; ThermoFisher), 0.1 % BSA (RRID: J64248.AE; ThermoFisher), 50 μg/mL apotransferrin (RRID: T2036–100 MG; Sigma), 5 μg/mL insulin (RRID: H0888–1G; Sigma), 30 nM sodium selenite (RRID: S5261–10G; Sigma), 10 nM D-biotin (RRID: B4639–100 MG; Sigma), 10 nM hydrocortisone (RRID: H0888–1G; Sigma), 10 ng/mL PDGF (RRID: P3326; Sigma), and 10 ng/mL bFGF (RRID: F0291; Sigma)]. Recently, we have seen that OPCs isolated by this procedure are highly pure and apparently that OPCs are free of either GFAP-positive astrocytes or Iba-1-positive microglia [6]. During treatment, OPCs were cultured in OPC medium in the absence of PDGF and bFGF and treated with different concentrations of wtTIDM and mTIDM for different time periods as mentioned for particular experiments.

### Real-time PCR analysis

Total RNA was isolated from OPCs using the QIAGEN RNeasy kit following the manufacturer’s protocol. The isolated RNA was reverse transcribed into cDNA, and real-time PCR was performed using the following primers:

MBP: Sense: 5^′^ - TGGAGAGATTCACCGAGGAGA −3′
Antisense: 5′ - TGAAGCTCGTCGGACTCTGAG −3 ′
PLP: Sense: 5 ′- CTTCCCTGGTGGCCACTGGATTGT −3′
Antisense: 5′ - CCGCAGATGGTGGTCTTGTAGTCG −3′
MOG: Sense: 5′ - CCTCTCCCTTCTCCTCCTTC −3′
Antisense: 5′ - AGAGTCAGCACACCGGGGTT −3′
GAPDH: Sense: 5′ - CAGGGGATGATCATGGCTTCTCC −3 ′
Antisense: 5′ - GATGCTCACAAGAGCCCCGTTAGC −3′

Real-time PCR was carried out in a QuantStudio 3 detection system (Thermo Fisher Scientific) using the SYBR green real-time kit obtained from Quantabio. Cycle threshold crossing point (*C*_*t*_) values for each sample were obtained from the software, and data were analyzed using the 2^−ΔΔCt^ method as described previously [17, 18].

### Western blot

It was performed as described before [6, 19, 20]. Briefly, equal amounts of proteins were electrophoresed in 10 % or 12 % polyacrylamide-SDS gels in 1X SDS running buffer. Proteins were transferred to 0.45 μm nitrocellulose membranes under wet conditions. Membranes were blocked for 1 h with Li-Cor blocking buffer, incubated with primary antibodies overnight at 4 ° C under shaking conditions, washed, incubated with IR-dye labeled secondary antibodies at room temperature, washed and visualized with the *Odyssey Infrared Imaging System (Li-Cor)*. Blots were converted to binary, analyzed using ImageJ (NIH) and normalized to the loading control (β-actin).

### Immunostaining

Cells were washed three times with 1X PBS, fixed in 4 % paraformaldehyde for 10 min or with chilled methanol (RRID: A412–500; ThermoFisher, Waltham, MA) overnight, washed again with 1X PBS, and incubated first with primary antibodies followed by Alexa Fluor 488- or Alexa Fluor 647-conjugated Affinity Pure Donkey secondary antibodies (Jackson ImmunoResearch) as described earlier [21–23]. For details on primary antibodies, please see Table 1. After secondary antibody incubation, coverslips were rinsed in 1X PBS, mounted on slides in Fluoromount *(Sigma)* and imaged using an *Olympus BX41* fluorescent microscope equipped with a *Hamamatsu ORCA-03G* camera. Immunostaining of tissue was performed by fixing the brains in 4 % paraformaldehyde followed by 30 % sucrose [24, 25]. Tissue was sectioned every 40 μm on a Leica Cryostat CM3050 S and kept in cryoprotectant. DAPI was added at the final step of washing of secondary antibodies for staining nuclei.

### Cell counting

Counting analysis was performed using the Olympus Micro-suite V software with the help of touch counting module as described [26, 27]. After acquiring images under 20× objective lens, images were further analyzed as follows. Before counting cells, entire Image area was calibrated with the help of a rectangular box available in the touch counting panel. Once the area of the image was measured, touch counting program was applied to count number of fluorescent signals. Next, the total number of signals in a given area was divided by the total area of the image and presented as number of cells per square millimeter unit.

### Mean fluorescence intensity (MFI) calculation

MFI was calculated using ImageJ as described before [17, 18, 28]. Briefly, to calculate the MFI of a whole image, the average pixel value of the whole frame was recorded followed by selecting a region of the image with no fluorescence from tissue or cell using the rectangle tool for measuring background intensity. Then true MFI of the image was determined by subtracting background intensity from the whole image intensity.

### Blinding and statistical analysis

All Western blot, real-time PCR, and immunohistochemical analyses were performed in a blinded manner. Laboratory personnel only knew about ID of each sample. For animal experiments, five mice were randomly included in each group. The sample size was similar to those reported in previous publications [29, 30] and is mentioned in figure legends. All cell culture experiments were repeated at least three times. Results were statistically analyzed using GraphPad Prism 10.1.1. Values are expressed as either mean ± SD or mean ± SEM. One-way ANOVA followed by Tukey’s multiple comparisons was performed for statistical analyses among multiple groups. For analyzing maturation of OPCs isolated from WT and TLR2^−/−^ mice, two-way ANOVA was performed followed by Tukey’s post hoc test. Normality assumptions were tested before applying ANOVA. The criterion for statistical significance was p<0.05.

## Results

### Effect of wtTIDM on the maturation of OPCs into OLs

**Figure 1:**
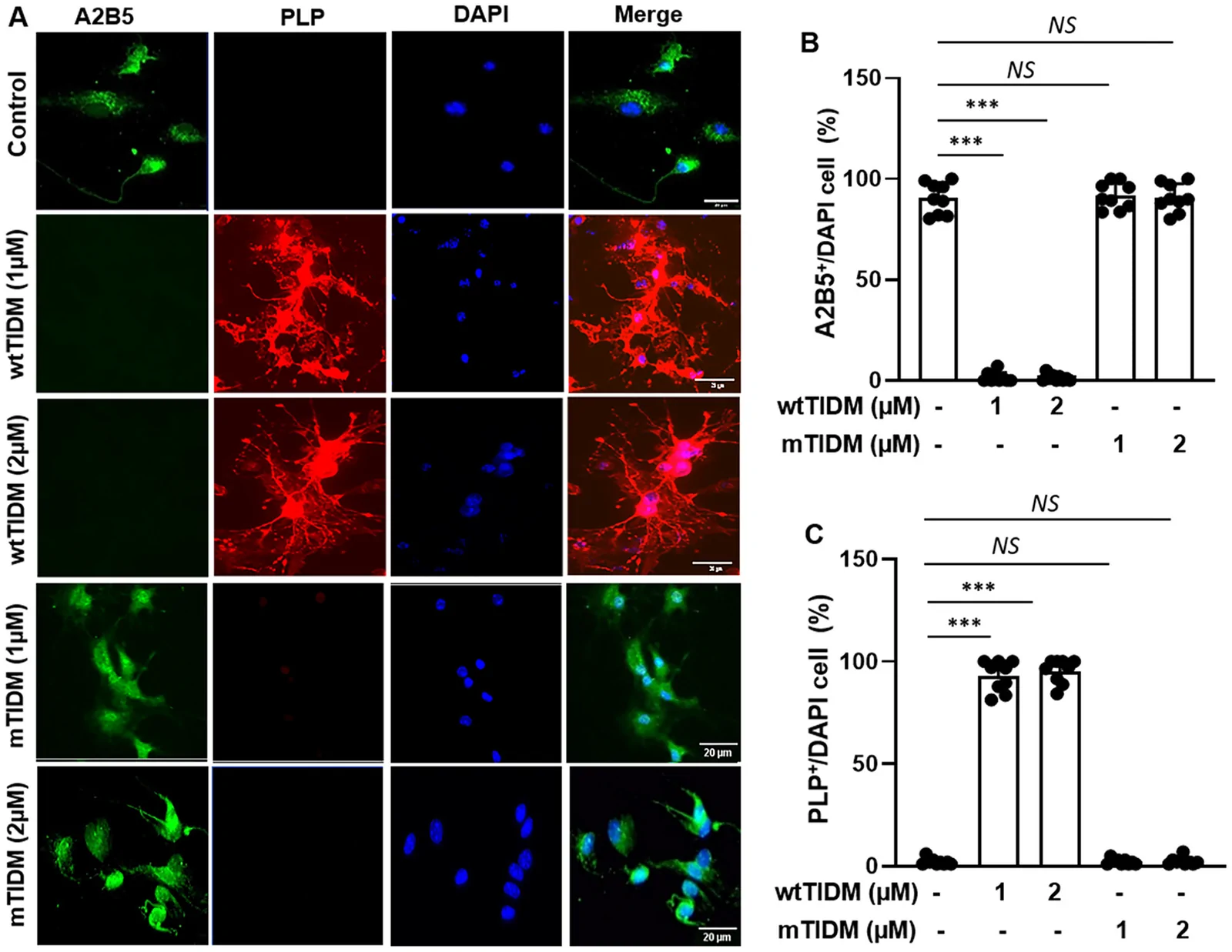
Effect of TIDM peptides on the maturation of OPCs into OLs. OPCs were isolated from P1-P2 neonatal pups, cultured in OPC media for 4 days *in vitro* (DIV) followed by treatment with wtTIDM and mTIDM peptides in the absence of FGF and PDGF. A) After 18 h of treatment, cells were double-labeled for PLP (red) and A2B5 (green). Nuclei were stained with DAPI (blue). Results represent three independent experiments. Quantification of A2B5^+^ (B) and PLP + (C) cells from 9 fields (3 fields per experiment) per group was presented as the percentage of total cells (DAPI^+^). One-way ANOVA followed by Tukey’s multiple comparison test was used for statistical analysis. *** p<0.001; NS, not significant.

**Figure 2:**
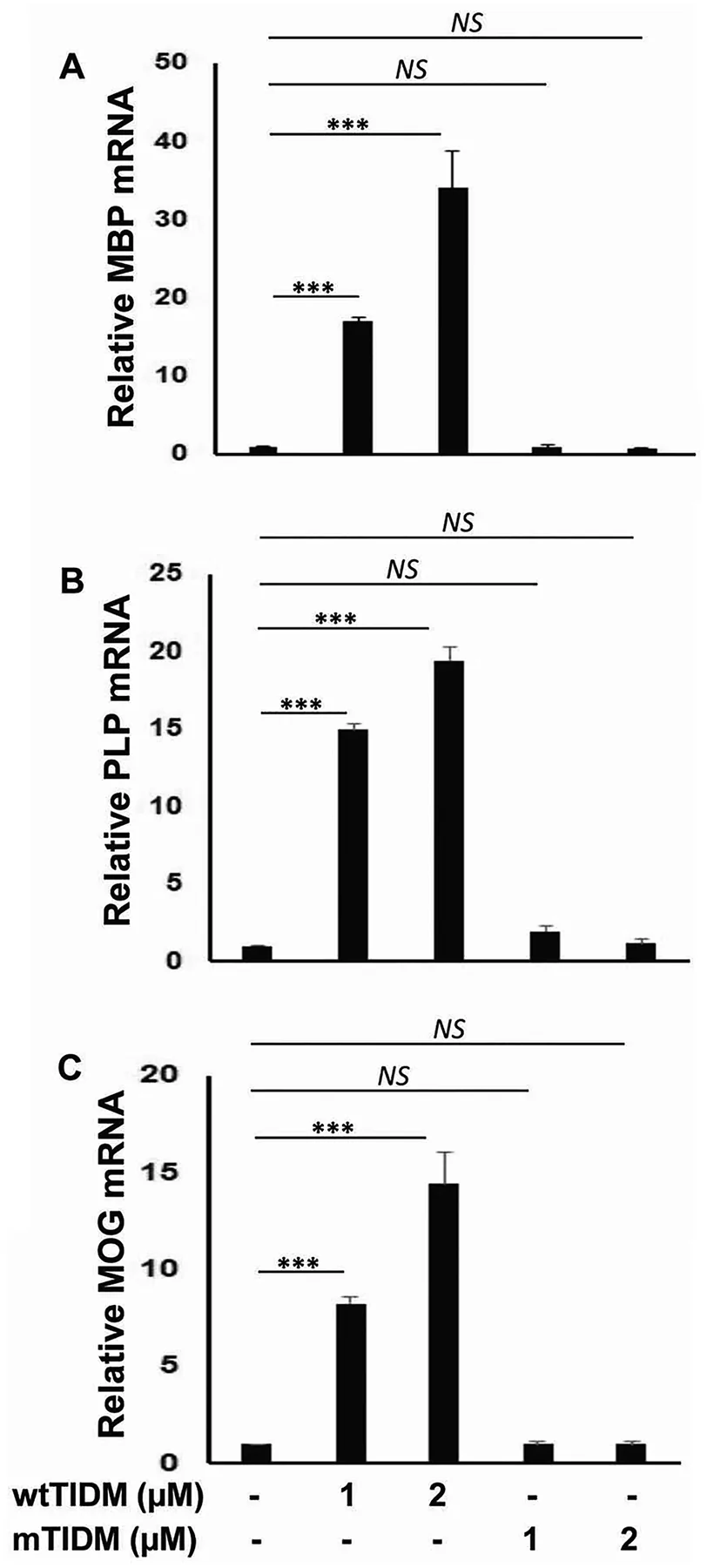
Stimulation of myelin-specific genes by wtTIDM in OPCs. OPCs were treated with different concentrations of wtTIDM and mTIDM for 5 h followed by monitoring the mRNA expression of MBP (A), PLP (B) and MOG (C) by real-time PCR. Results are mean ± SD of three different experiments. One-way ANOVA followed by Tukey’s multiple comparison test was used for statistical analysis. ***p<0.001; NS, not significant.

OPCs are switched towards new myelinating OLs during remyelination. A2B5, a cell surface ganglioside epitope, is considered as a reliable marker of OPCs. According to Figarella-Branger et al. [31], A2B5 is required probably to support the self-renewal property of OPCs. Since PLP is a marker of OLs, downregulation of A2B5 along with subsequent upregulation of PLP is therefore used to examine the generation of OLs from OPCs. Primary mouse OPCs isolated from WT mice were treated with different doses of wtTIDM and mTIDM for 18 h followed by immunostaining with PLP and A2B5. We found that wtTIDM at both 1 and 2 μM concentrations upregulated the level of PLP with strong inhibition of A2B5 (Figure 1A). To confirm the finding further, we performed cell counting that clearly showed marked reduction in A2B5^+^ (Figure 1B) along with strong increase in PLP^+^ (Figure 1C) cells. To confirm the findings further, we examined the mRNA expression of MBP, PLP and MOG (markers of OLs) by real-time PCR. Consistent to immunostaining results, wtTIDM, but not mTIDM, increased the mRNA expression of *MBP* (Figure 2A), *PLP* (Figure 2B) and *MOG* (Figure 2C) in OPCs, indicating that wtTIDM, but not mTIDM, is capable of inducing maturation of OPCs to OLs.

### The wtTIDM induces the maturation of OPCs into OLs in TLR4^−/−^, but not TLR2^−/−^, OPCs

**Figure 3:**
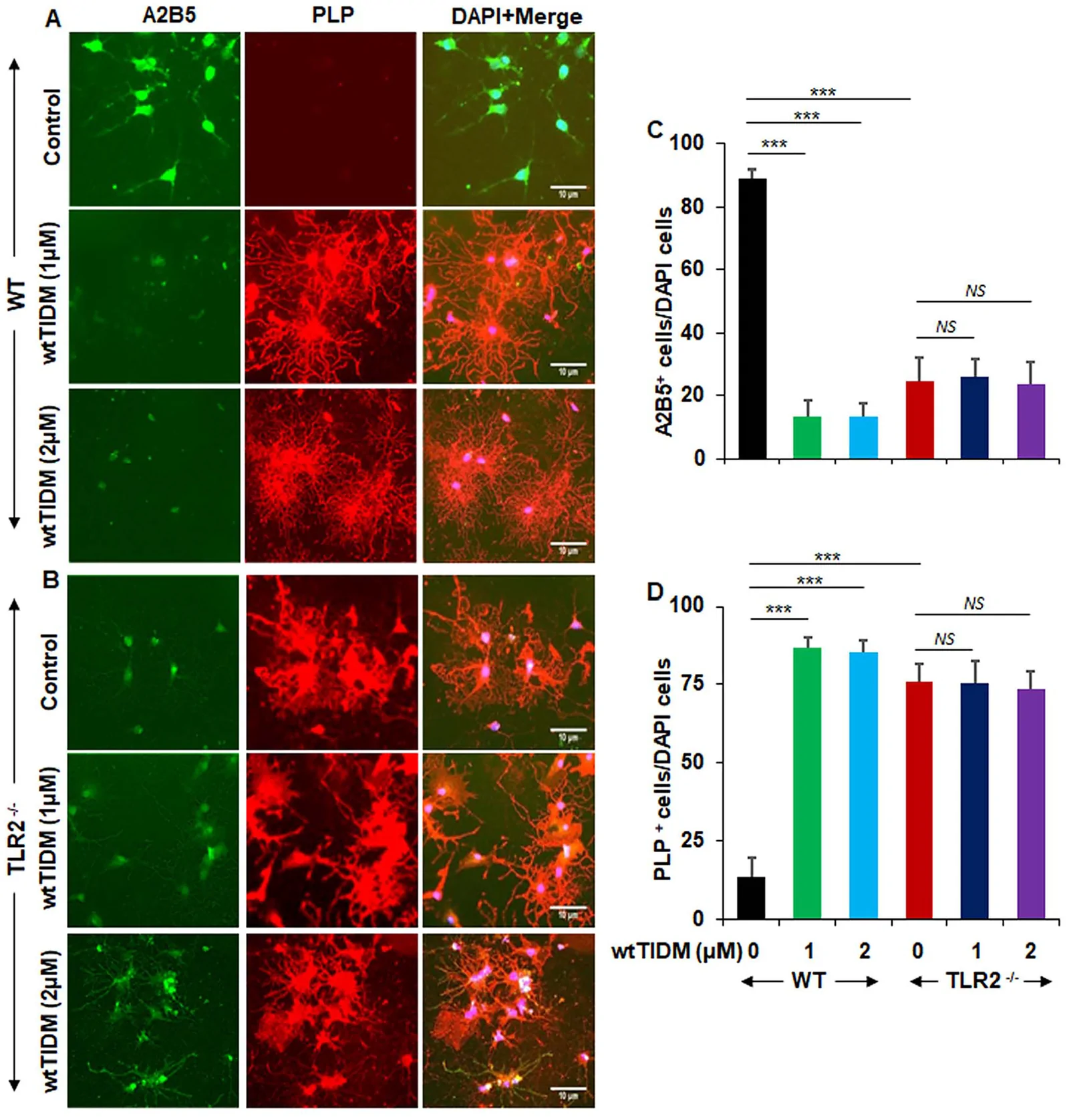
In the absence of TLR2, wtTIDM peptide remained ineffective in stimulating the maturation of OPCs into OLs. OPCs were isolated from both WT and TLR2^−/−^ P1-P2 neonatal pups, cultured in OPC media for 4 days *in vitro* (DIV) followed by treatment with wtTIDM peptides in the absence of FGF and PDGF. After 18 h of treatment, cells were double-labeled for PLP (red) and A2B5 (green) (A, WT; B, TLR2^−/−^). Nuclei were stained with DAPI (blue). Results represent three independent experiments. Quantification of A2B5^+^ (C) and PLP + (D) cells from 9 fields (3 fields per experiment) per group was presented as the percentage of total cells (DAPI^+^). Results were statistically analyzed by two-way ANOVA followed by Tukey’s post hoc test.***p<0.001; NS, not significant.

Since wtTIDM is known to bind TLR2, but not other TLRs [9], to understand the specificity of this promyelinating function of wtTIDM, OPCs isolated from TLR2^−/−^ and TLR4^−/−^ mice were treated with wtTIDM peptide. After 24 h of treatment, OPCs were double-labeled with antibodies against PLP and A2B5. As evident from Figure 3A–D, the knockdown of TLR2 alone was sufficient to significantly increase the levels of PLP^+^ OLs and decrease the number of A2B5^+^ OPCs as compared to control WT OPCs, indicating that TLR2 is a negative regulator of OPC maturation. However, treatment with wtTIDM peptide remained ineffective to further upregulate PLP and downregulate A2B5 in TLR2^−/−^ OPCs, indicating that in the absence of TLR2, the wtTIDM is unable to stimulate the maturation of OPCs to OLs.

**Figure 4:**
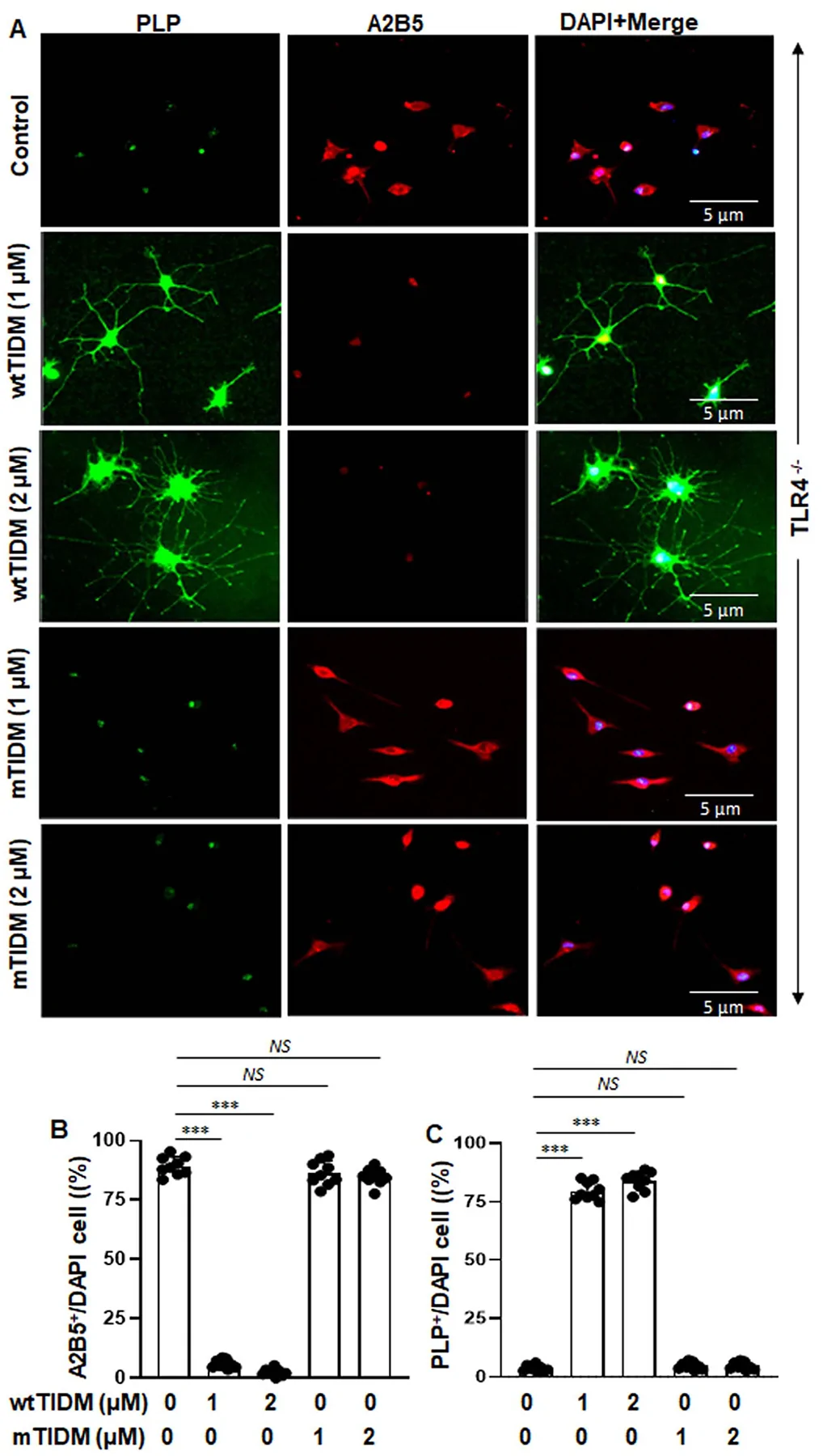
The wtTIDM does not require TLR4 to induce the maturation of OPCs into OLs. OPCs were isolated from TLR4^−/−^ P1-P2 neonatal pups, cultured in OPC media for 4 days *in vitro* (DIV) followed by treatment with wtTIDM and mTIDM peptides in the absence of FGF and PDGF. A) After 18 h of treatment, cells were double-labeled for PLP (red) and A2B5 (green). Nuclei were stained with DAPI (blue). Results represent three independent experiments. Quantification of A2B5^+^ (B) and PLP + (C) cells from 9 fields (3 fields per experiment) per group was presented as the percentage of total cells (DAPI^+^). One-way ANOVA followed by Tukey’s multiple comparison test was used for statistical analysis. *** p<0.001; NS, not significant.

On the other hand, in contrast to the knockdown of TLR2, the absence of TLR4 alone did not augment the number of PLP^+^ OLs and reduce the number of A2B5^+^ OPCs (Figure 4A–C), indicating that dissimilar to TLR2, TLR4 is not a negative regulator of OPC maturation. However, similar to WT OPCs (Figure 3A, C and D), the wtTIDM peptide markedly increased the number of PLP^+^ OLs and decreased the number of A2B5^+^ OPCs in TLR4^−/−^ OPCs (Figure 4A–C). Although TLR4 is an important TLR, our results clearly indicate that wtTIDM does not require TLR4 for stimulating the maturation of OPCs to OLs.

### Intranasal administration of wtTIDM stimulates remyelination in the corpus callosum of cuprizone-intoxicated mice

**Figure 5:**
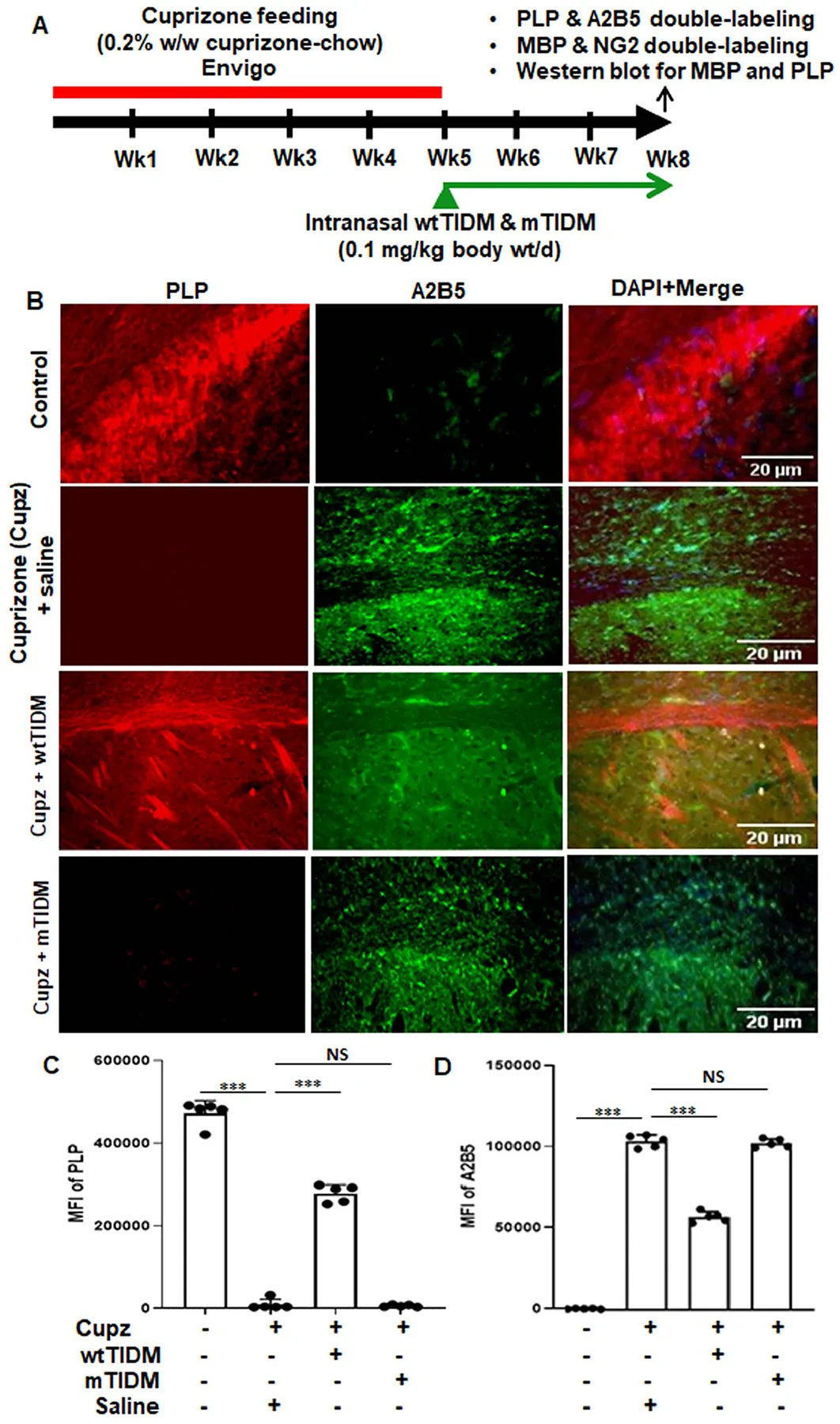
Effect of intranasal wtTIDM and mTIDM peptides on the maturation of OPCs in the corpus callosum of cuprizone (Cupz)-intoxicated mice. Eight-weeks-old C57/BL6 male mice (n=5 per group) received standard rodent chow mixed with 0.2 % (w/w) Cupz (Envigo). After 5 weeks of Cupz intoxication, mice were provided regular chow and treated with wtTIDM (0.1 mg/kg body wt/d) and mTIDM (0.1 mg/kg body wt/d) peptides intranasally once daily (A). Since TIDM peptides were solubilized in saline, cuprizone-insulted mice received 2.5 μL of saline intranasally as vehicle control. After 3 weeks of treatment, corpus callosum sections were double-labeled with antibodies against PLP and A2B5 (B) and quantification of mean fluorescence intensity (MFI) of PLP (C) and A2B5 (D). Two sections of each of 5 mice per group were used for MFI calculation. The section MFIs were averaged per mouse first, and statistical testing was done at the mouse level. Results are mean ± SEM of 5 mice per group. One-way ANOVA followed by Tukey’s multiple comparison test was used for statistical analysis. ***p<0.001; NS, not significant.

Since cuprizone-insulted mice exhibit demyelination in the brain particularly in the myelin-rich corpus callosum [32], these mice are generally used to monitor remyelinating efficacy of different drugs *in vivo* in the brain. Therefore, after 5 weeks of cuprizone feeding, groups of mice (n=5) were treated with 0.1 mg/kg body weight of wtTIDM and mTIDM daily via intranasal route. Please see the schematic presentation of the experiment (Figure 5A). After 3 weeks of wtTIDM/mTIDM treatment, the maturation of OPCs to OLs was monitored in the corpus callosum by double-labeling of corpus callosum sections for PLP & A2B5. As expected, widespread demyelination was observed in the corpus callosum of cuprizone-intoxicated mice in comparison to control mice (Figure 5B). The corpus callosum of cuprizone-insulted mice was full of A2B5^+^ OPCs and almost devoid of PLP^+^ OLs and myelin (Figure 5B–D). However, 3 weeks of wtTIDM treatment markedly increased the level of PLP (Figure 5B and C) and decreased the level of A2B5 (Figure 5B and D) in the corpus callosum of cuprizone-intoxicated mice, indicating that intranasal wtTIDM is capable of stimulating remyelination in the brain of cuprizone-challenged mice. On the other hand, such increase in PLP and decrease in A2B5 were not found in mTIDM-treated cuprizone-challenged mice (Figure 5B–D).

**Figure 6:**
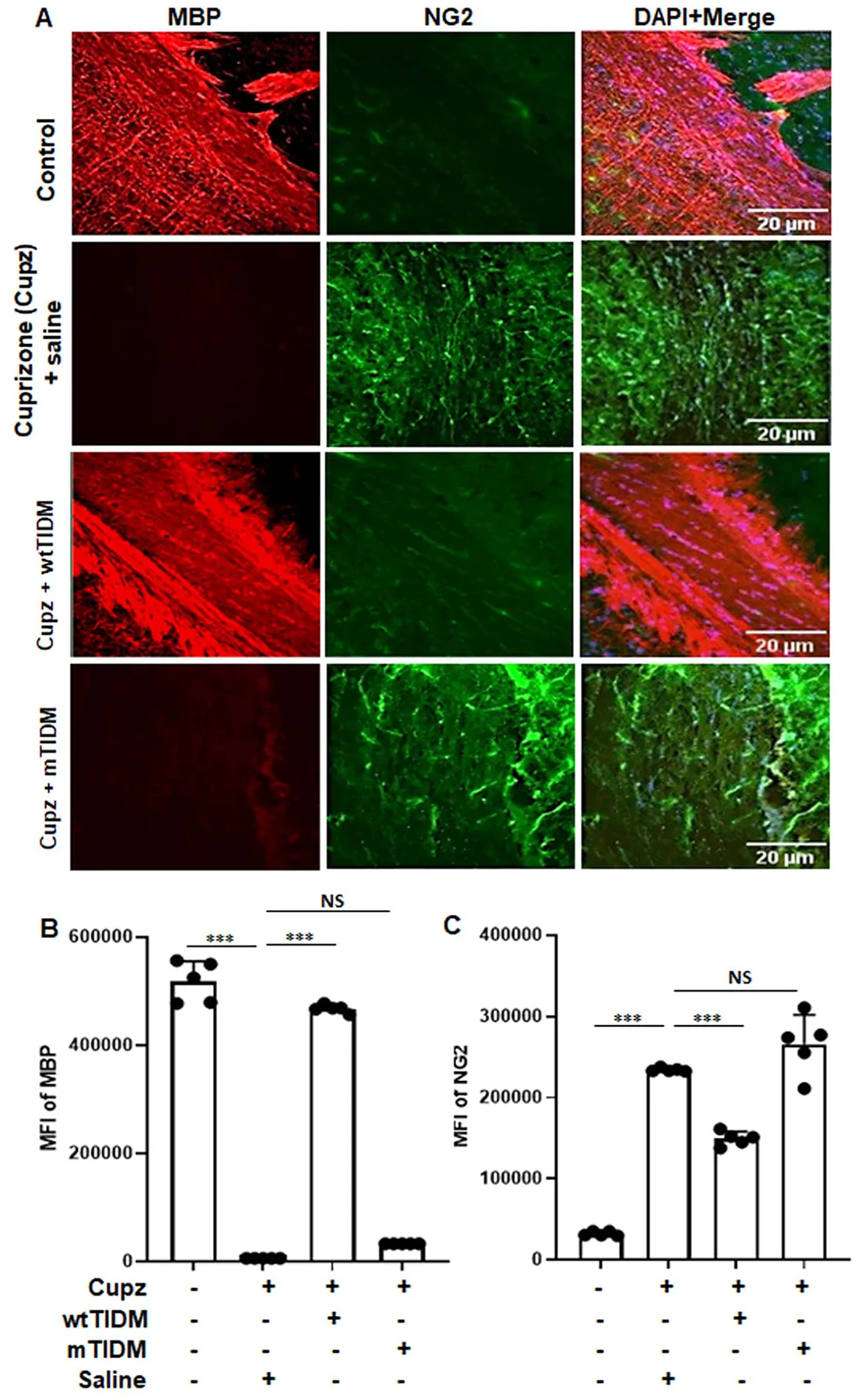
Effect of intranasal wtTIDM and mTIDM peptides on the maturation of OPCs in the corpus callosum of cuprizone (Cupz)-intoxicated mice. Eight-weeks-old C57/BL6 male mice (n=5 per group) received standard rodent chow mixed with 0.2 % (w/w) Cupz (Envigo). After 5 weeks of Cupz intoxication, mice were provided regular chow and treated with wtTIDM (0.1 mg/kg body wt/d) and mTIDM (0.1 mg/kg body wt/d) peptides intranasally once daily. Since TIDM peptides were solubilized in saline, cuprizone-insulted mice received 2.5 μL of saline intranasally as vehicle control. After 3 weeks of treatment, corpus callosum sections were double-labeled with antibodies against MBP and NG2 (A) followed by quantification of mean fluorescence intensity (MFI) of MBP (B) and NG2 (C). Two sections of each of 5 mice per group were used for MFI calculation. The section MFIs were averaged per mouse first, and statistical testing was done at the mouse level. Results are mean ± SEM of 5 mice per group. One-way ANOVA followed by Tukey’s multiple comparison test was used for statistical analysis. ***p<0.001; NS, not significant.

To confirm this finding further, corpus callosum sections were immunostained with antibodies against MBP and NG2. Similar to PLP, remarkable loss of MBP was seen in the corpus callosum of cuprizone-fed mice as compared to normal mice (Figure 6A and B). On the other hand, as expected, NG2-positive OPCs were recruited to the corpus callosum of cuprizone-insulted mice (Figure 6A and C). However, intranasal treatment with wtTIDM, but not mTIDM, led to upregulation of MBP and downregulation of NG2 in the corpus callosum of cuprizone-challenged mice (Figure 6A–C).

**Figure 7:**
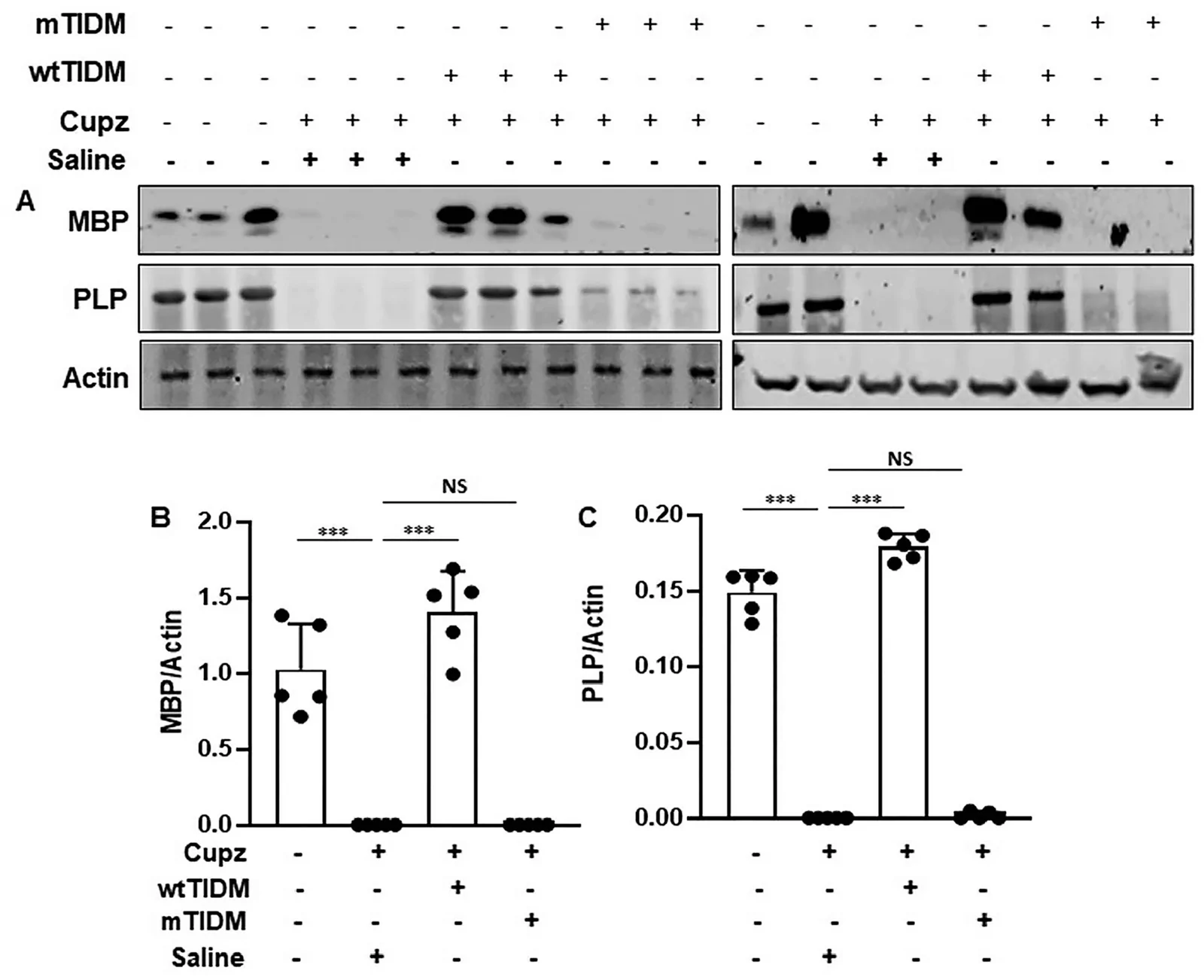
Effect of intranasal wtTIDM and mTIDM peptides on remyelination in the corpus callosum of cuprizone (Cupz)-intoxicated mice. Eight-weeks-old C57/BL6 male mice (n=5 per group) received standard rodent chow mixed with 0.2 % (w/w) Cupz (Envigo). After 5 weeks of Cupz intoxication, mice were provided regular chow and treated with wtTIDM (0.1 mg/kg body wt/d) and mTIDM (0.1 mg/kg body wt/d) peptides intranasally once daily. Since TIDM peptides were solubilized in saline, cuprizone-insulted mice received 2.5 μL of saline intranasally as vehicle control. After 3 weeks of treatment, corpus callosum extracts were immunoblotted for MBP and PLP (A). Actin was run as a loading control. Bands were scanned and values (B, MBP/Actin; C, PLP/Actin) presented. Results are mean ± SEM of 5 mice per group. One-way ANOVA followed by Tukey’s multiple comparison test was used for statistical analysis. ***p<0.001; NS, not significant.

For further confirmation of remyelinating efficacy of wtTIDM, protein levels of MBP and PLP were monitored in corpus callosum by Western blot. Similar to immunofluorescence results, marked reduction of MBP and PLP was seen in the corpus callosum of cuprizone-fed mice in comparison to control mice (Figure 7A–C). However, intranasal wtTIDM increased the levels of both MBP and PLP in cuprizone-intoxicated mice, which was not seen in case of mTIDM treatment (Figure 7A–C). These results suggest that wtTIDM, but not mTIDM, is capable of stimulating remyelination in the brain of a mouse model of demyelination.

## Discussion

Myelin is a lipid rich plasma membrane [4, 33], which forms manifold concentric coatings around the axon for preserving electrical conduction through a healthy neuron [34]. However, in demyelinating conditions, the ionic conduction through the neuron is reduced, ultimately leading to axonal loss. Therefore, demyelination is considered as an important pathological hallmark in several neuroinflammatory disorders including MS [35], Devic’s disease [36], progressive multifocal leukoencephalopathy [37], Krabbe disease [38], neuromyelitis optica [39], and X-Adrenoleukodystrophy [40, 41]. Since OLs are the only myelin-synthesizing cells in the CNS, the death or dysfunction of OLs is considered as the major cause of demyelination [38, 42–47].

Dissimilar to most progenitor cells, OPCs stay throughout life as adult [48]. According to Elbaz and Popko [49], self-renewing OPCs are capable of differentiating into newly formed myelinating OLs for maintaining myelin plasticity. Accordingly, lost myelin may be regenerated during remyelination, thus preventing axonal degeneration and restoring function. It has been shown that defective maturation of OPCs into OLs is one of the important primary mechanisms of demyelination [50–52]. In chronic active lesions, Boyd et al. have shown that deficiency of OPC within lesions either due to impaired recruitment and/or proliferation is another cause of remyelination failure [53]. Although remyelination is a natural regenerative process, which is believed to prevent neurodegeneration and restore function in human MS lesions, Chang et al. [54] and Kuhlmann et al. [55] identified a block in OPC differentiation into oligodendrocytes. Therefore, describing underlying mechanisms behind defective OPC maturation and developing an effective neuroprotective therapeutic approach to achieve remyelination are important areas of research.

Recently, we have developed wtTIDM peptide that inhibits microglial activation induced by lipoteichoic acid and fibrillar Aβ, tau and *α*-syn [8, 9, 13]. In contrast, wtTIDM peptide does not interfere with LPS-, IL-1β-, polyIC-, MPP^+^ -, flagellin-, and CpG DNA-mediated activation of microglia [9]. This is because wtTIDM is specific for TLR2 and fibrillar Aβ, tau and *α*-syn activate microglia via TLR2 [8, 9, 13, 56]. It has been shown that TLR2 is expressed in OLs in MS lesions [12] and that TLR2 ligands as well as hyaluronan hold OPCs in an immature state via TLR2 [12]. Therefore, we examined the efficacy of wtTIDM in promoting the maturation of OPCs. The results clearly demonstrate that low dose wtTIDM markedly stimulates the maturation of OPCs to OLs. MBP, PLP and MOG are considered as specific markers of matured OLs, and it is nice to see that micromolar concentrations of wtTIDM readily upregulate MBP, PLP and MOG in OPCs. For checking the specificity, when TLR2^−/−^ and TLR4^−/−^ OPCs were used for treatment, wtTIDM stimulated the maturation in TLR4^−/−^, but not TLR2^−/−^, OPCs, clearly delineating the involvement of TLR2 in wtTIDM-mediated OPC maturation. This also suggests that wtTIDM will not function if TLR2 is not present.

In human MS, chronic demyelination often becomes recalcitrant to simple differentiation-inducing agents, indicating that in the chronically demyelinated CNS, inducing differentiation alone sometimes fails to support full repair processes if the underlying inflammatory “milieu” is not addressed. Although we have not analyzed microglial activation here, glial inflammation plays an important role in the pathogenesis of demyelination in MS [57, 58]. Several studies from CNS tissues of MS and experimental autoimmune encephalomyelitis, an animal model of MS, have suggested that proinflammatory cytokines, chemokines, nitric oxide, etc. released activated glial cells and macrophages create a conducive environment for demyelination and a hostile environment for remyelination in MS [2, 58–60]. Therefore, chronic neuroinflammation and demyelination and/or failure to remyelination are important contributors of disability among patients with MS [57, 58, 61–64]. However, microglia are rich in TLR2 and MyD88 and in several studies [8, 9, 13], we have already described that wtTIDM is capable of inhibiting microglial inflammation. Since wtTIDM is also capable of promoting the maturation of OPCs, it may be a valuable tool for encouraging remyelination in an inflamed CNS.

While many molecules exhibit function in cell cultures, very few cross the blood-brain barrier (BBB) and show efficacy *in vivo* in the brain. However, wtTIDM contains the antennapedia homeodomain tag at the N-terminus, which helps the wtTIDM to enter into the cells and cross the BBB [8, 9]. Therefore, after intranasal administration, wtTIDM enters into different parts of the brain of diseased as well as normal mice. While by using infrared scanning and electrospray ionization-coupled mass spectrometry, Rangasamy et al. [9] have reported the entry of intranasally delivered wtTIDM into the hippocampus of 5XFAD mice, Dutta et al. [8] have detected wtTIDM in frontal cortex, midbrain and cerebellum of normal B6C3 mice by infrared scanning after intranasal administration. Accordingly, after 3 weeks of treatment, the wtTIDM stimulated the maturation of OPCs and increased remyelination in corpus callosum of cuprizone-intoxicated mice. Our conclusions are based on the following. *First*, the wtTIDM, but not mTIDM, peptide treatment increased the level of both PLP and MBP (markers of OLs and myelin) in the corpus callosum of cuprizone-intoxicated mice. *Second*, levels of both A2B5 and NG2 (markers of OPCs) decreased after treatment with wtTIDM, but not mTIDM, peptide. TLR2 is known to function via both MyD88-dependent and – independent pathways [65, 66] and the real strength of our finding is that TIDM peptide targets only the MyD88-dependent induced TLR2 signaling pathway without inhibiting basal TLR2 activity.

Although until now, there is no FDA-approved remyelination-specific drug, recent studies have identified several ones for clinical or preclinical development. For example, a selective M1 muscarinic receptor antagonist (PIPE-307) [67] and a synthetic peptide (FTX-101) designed to disrupt the Plexin A1/Neuropilin 1 (NRP1) receptor complex [68] are being developed for remyelination in MS patients based on their potential on stimulating the maturation of OPCs. While these drugs are targeting either M1 muscarinic G protein-coupled receptor or semaphorin transmembrane receptor, wtTIDM is targeting a specific pattern recognition receptor (TLR2) in the innate immune system [8, 9].

In summary, here, we demonstrate that selective inhibition of TLR2-to-MyD88 interaction by wtTIDM stimulates the differentiation of OPCs to OLs via TLR2, but not TLR4, and that after intranasal administration, the wtTIDM increases the differentiation of OPCs and promotes remyelination in the corpus callosum of cuprizone mouse model of demyelination. Therefore, specific blocking of the TLR2-MyD88 pathway with the synthetic peptide wtTIDM may be considered for remyelination in MS and other demyelinating conditions.

## Figures and Tables

**Table 1:** Antibodies used for the study.

Protein	Source	Catalogue no.	Application/Dilution	Host species
Proteolipid protein (PLP)	Abcam	AB28486	WB/1:1,000 IHC/1:500	Rabbit
Myelin basic protein (MBP)	Santa cruz	SC-271524	WB/1:1,000 IHC/1:500	Mouse
NG2 chondroitin sulfate proteoglycan	EMD millipore	AB5320	IFC/1:500	Rabbit
A2B5	Abcam	AB53521	IFC/1:500	Mouse
Beta-actin	Abcam	AB6276	WB:1/10,000	Mouse

WB, western blot; IFC, immunofluorescence; IHC, immunohistochemistry.

## Data Availability

Data will be made available on request.
